# First International Electronic Conference on Medicinal Chemistry (ECMC-1)

**DOI:** 10.3390/ph9010014

**Published:** 2016-03-11

**Authors:** Annie Mayence, Jean Jacques Vanden Eynde

**Affiliations:** 1 Haute Ecole Provinciale de Hainaut Condorcet, 7330 Saint-Ghislain, Belgium; annie.mayence@condorcet.be; 2 University of Mons-UMONS, 7000 Mons, Belgium

**Keywords:** biological and therapeutic targets, biomolecules, drug development, drug discovery, medicinal chemistry, pharmacological evaluation

## Abstract

The first International Electronic Conference on Medicinal Chemistry, organized and sponsored by MDPI AG, publisher, and the Journal *Pharmaceuticals,* took place in November 2015 on the SciForum website. More than 200 authors from 18 countries participated in the event and was attended by 25,000 visitors who had the opportunity to browse among 55 presentations, keynotes, and videos. A short description of some works presented during that scientific meeting is disclosed in this report.

## 1. Aim and Scope

The first International Electronic Conference on Medicinal Chemistry (www.sciforum.net/conference/ecmc-1) was organized by the journal *Pharmaceuticals*, published by MDPI AG. It took place on the Internet on 2–27 November 2015. The goal of the organizers was to invite researchers involved in the field of drug discovery and drug development to present their recent work to the scientific community and to share their results with academic and industrial groups from all over the world. The procedure selected for this first edition was constituted by four essential steps: (i) online submission of an abstract consisting of a brief description of the topic covered by the authors; (ii) evaluation of the scientific adequacy of the subject with the scope of the conference; (iii) online submission of a full presentation under the form of a slide show; (iv) evaluation of the scientific and general qualities of that presentation. After selection, 55 communications, including 7 keynotes and videos, were posted on the website. A brief summary of some of them is presented hereafter.

## 2. Presentations

### 2.1. Consideration of the Stereochemical Features of Compounds in QSAR Models. 2D+0.X Molecular Descriptors (A001)

Adlen Mouats ^1,2,^*, Victor E.Kuz’min ^2^ and Anatoliy G. Artemenko ^2^

^1^ I.I. Mechnikov Odessa National University, Dvoryanska Street 2, Odessa 65002, Ukraine

^2^ A.V. Bogatsky Physico-Chemical Institute of the National Academy of Sciences of Ukraine, Lyustdorfska doroga Street 86, Odessa 65080, Ukraine

* Correspondence: nandoura92@gmail.com

In chemoinformatics, stereochemical attributes are commonly taken into account only by direct description of spatial structures via 3D-QSAR approaches which are applied for one fixed conformer of each molecule. That can be undesirable if we do not know the spatial structure of the molecule interacting with a biological target. In this study, we show how to solve this problem in terms of simplex representation of the molecular structure (SiRMS).

In the SiRMS approach, every molecule is represented as a system of different simplexes (tetratomic fragments with fixed composition and structure). The advantages of that approach are the absence of “molecular alignment” problems, consideration of different physical-chemical properties of atoms (e.g., charge and lipophilicity), the high adequacy and good interpretability of obtained models, *etc*. In this study, all molecular fragments which do not determine stereochemistry of a molecule are described in terms of 2D molecular representation (structural formula). Structural elements which determine molecular stereoisomerism are described by respective 3D chiral conformation-independent simplexes.

**Figure:**
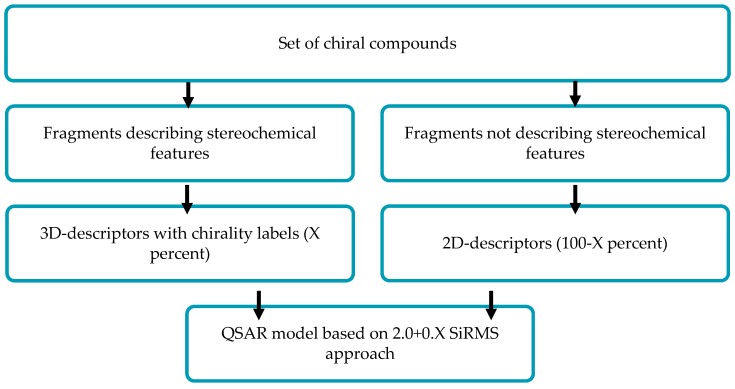


It should be noted that chiral simplexes allow us to describe the molecular system of any stereochemical complexity. In the proposal (2.0+0.X), D-QSAR approach parameter (0.X) is determined by the ratio of 2D achiral and 3D chiral simplexes.

### 2.2. Chemical and Biological Characterizations of an Effective Bimodal Probe to Target Apoptosis (A002)

Mario Dentamaro ^1,2,3,^*, François Lux ^2^, Sylvie Montante ^1^, Luce Vander Elst ^1,3^, Robert N. Muller ^1,3^, Olivier Tillement ^2^ and Sophie Laurent ^1,3,^*

^1^ University of Mons, Department of General, Organic and Biomedical Chemistry, Laboratory of NMR and Molecular Imaging, Avenue Maistriau, 19, B-7000 Mons, Belgium

^2^ University Claude Bernard-Lyon 1, Institut Lumière Matière, FENNEC Team, UMR CNRS-5305, F-69622 Villeurbanne Cedex, France

^3^ CMMI-Center of Microscopy and Molecular Imaging, Rue Adrienne Bolland, 8, B-6041 Gosselies, Belgium

* Correspondence: mario.dentamaro@umons.ac.be (M.D.); sophie.laurent@umons.ac.be (S.L.)

AGuIX^®^ nanoparticles are small platforms of polysiloxane developed for applications in medical imaging. These nanoparticles present on their surface several amine functions used for the fixation of Gd-DOTA ligands (Lux, F.; *et al. Angew. Chem. Int. Ed.* 2011, *50*, 1–6). These paramagnetic platforms have a diameter of less than 5 nm and a low transmetalation (Mignot, A.; *et al.* *Chem. Eur. J.* 2013, *19*, 6122–6136). Different studies have been achieved showing that they allow for the combining of multimodal and theranostic properties with a passive tumoral targeting observed by the EPR effect (enhanced permeation effect). Otherwise, their small size allows for a quick elimination by the kidney (Le Duc, G.; *et al.* *ACS Nano* 2011, *5*, 9566–9574).

Some studies have shown that a TLVSSL peptide has a high affinity for phosphatidylserine, a phospholipid overexposed on cells in the apoptosis process of death (Laumonier, C.; *et al.* *Technol. J. Biomol. Screen.* 2006, *11*, 537–545). The targeting of apoptotic cells is interesting for its efficiency of antitumoral therapy and for diagnosis of diseases related to this process. In this work, AGuIX^®^ nanoparticles have thus been grafted with this peptide and characterized.

AGuIX^®^ nanoparticles were grafted with the TLVSSL peptide by activation with EDC of carboxylic functions available on the nanoparticle surface. Furthermore, a previous addition of an optical dye allows for their applications in optical imaging. To increase the peptide mobility against the rigid platform, AGuIX nanoparticles were also grafted with peptides bound to a linker through an amide bond with 8-amino-3,6-dioxaoctanoic acid. Different physicochemical techniques such as PCS, fluorescence spectroscopy, HPLC, and proton relaxometry were used to characterize these platforms. Biological tests were performed to determine their affinity for apoptotic Jurkat cells.

Relaxometric studies by NMRD profiles confirm the increase of the rotational correlation time after the linking of the peptide and allow for the studying of the time stability of the platform. The biological efficiency of this novel bimodal agent to target apoptotic cells was evaluated via fluorescence microscopy on a lymphoblastic human T-cell line. *In vitro* cell apoptosis was chemically induced by incubation with campthothecin. Fluorescence microscopy on incubated cells with labeled and grafted-AGuIX nanoparticles allowed for the confirmation of the probe efficiency in targeting apoptosis.

**Figure:**
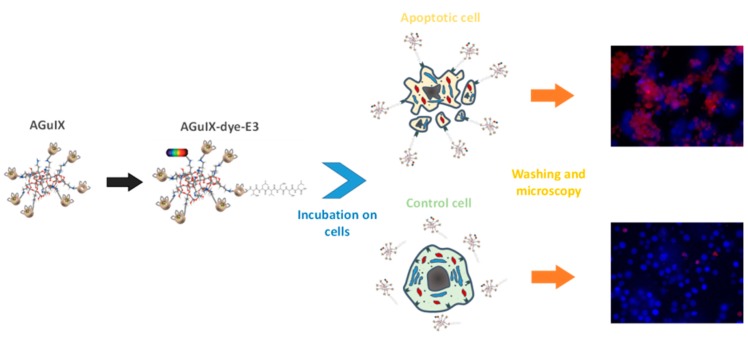


**Acknowledgments:** This work was performed with the financial support of F.R.S.-FNRS, the FEDER, the Walloon Region, the COST Action TD1004, the CMMI supported by the European Regional Development Fund of the Walloon region, the ARC, and UIAP programs. The French program ANR-12-RPIB-0010 Multimage is also acknowledged for its funding.

### 2.3. Biochemical Link between Chromogranin A and Cyclooxygenase-2 in Pheochromocytoma Pathology (A003)

Ana-Maria Stefanescu * and Sorina Schipor

National Institute of Endocrinology “C. I. Parhon”, Research Department – Biogenic Amines Lab, 34-38 Bd. Aviatorilor, 011863 Bucharest, Romania

* Correspondence: stefanescuam@yahoo.com

The precise biological function of elevated chromogranin A (CgA) in neuroendocrine and nonneuroendocrine neoplasms remains unclear (Connolly, R.; *et al.* *Endocrinology* 2007, *148,* 4310–4317). In a neuroendocrine tumor (NET)-derived cell line study, it was demonstrated that cyclooxygenase-2 (Cox-2) upregulates both CgA expression and bioactivity with implications of this polypeptide in neuroendocrine cancer (Salmenkiwi, K.; *et al.* *J. Clin. Endocrinol. Metable* 2001, *86*, 5615–5619; Lenders, J.W.M.; *et al.* *Lancet* 2005, *366*, 665–675).

In our study, we indirectly tested the link between Cox-2 and CgA in 15 patients that were clinically suspected of pheochromocytoma by comparison with 15 matched controls without endocrine dysfunction. Biochemical diagnosis of pheochromocytoma was realized by a differential assay of plasma free normetanephrines (NMNp)/free metanephrines (MNp) and by a plasma assay of CgA and Cox-2. All four parameters were assayed both in tumoral and normal subjects. We established statistically significant differences between all parameters assayed. Multiple regression showed important correlation coefficients between NMNp/CgA, NMNp/MNp, and CgA/Cox-2. Practically, we proved the traffic control of the noradrenergic metabolite NMNp by CgA and Cox-2. Using relative operating curve analysis (ROC), we were able to compare sensitivity and specificity of all four assayed parameters. Cox-2, CgA, and NMNp were proven to have the best sensitivity and a great specificity.

We conclude that Cox-2 can be used as a prediction marker in pheochromocytoma pathology together with CgA/NMNp/MNp.

**Acknowledgments:** This work was financially supported by PNCDI II Grant 42-101/2008.

### 2.4. Microwave-Assisted C–H Arylation of Quinazolin-4-one-type Precursors of Bioactive Heterocycles (A004)

Corinne Fruit *, Julien Godeau, Marine Harari, Sylvain Laclef, Vincent Levacher and Thierry Besson

Normandie Univ, COBRA, UMR 6014 & FR 3038; Univ Rouen; INSA Rouen; CNRS, IRCOF, 1 rue Tesnière, 76821 Mont Saint Aignan Cedex, France

* Correspondence: corinne.fruit@univ-rouen.fr

Our group was focused on the synthesis of C,N,S- or C,N,O-containing heterocyclic precursors of bioactive molecules able to modulate the activity of kinases involved to some extent in Alzheimer's disease (Schmitt, C.; *et al.* *ACS Med.* *Chem.* *Lett.* 2014, *5*, 963–967; Dehbi, O.; *et al.* *Eur. J. Med. Chem.* 2014, *80*, 352–363). Previous biological results inspired us to intensively study thiazoloquinazolin-4-one backbone, especially modulations of positions **1** and **2**. Following our effort for the construction of a broad range of substituted thiazoloquinazolin-4-one derivatives as potential kinase inhibitors (Foucourt, A.; *et al.* *Molecules* 2014, *19*, 15546–15571; Foucourt, A.; *et al.* *Molecules* 2014, *19*, 15411–15439; Deau, E.; *et al.* *Tetrahedron Letters* 2013, *54*, 3518–3521; Schmitt C.; *et al.* *ACS Med. Chem. Lett.* 2014, *5*, 963–967), modulation of position **3** was further explored. As an efficient and versatile approach in complex molecule synthesis that is palladium-catalyzed, C–H functionalization of heteroarenes represents an extremely attractive approach (Rossi, R.; *et al.* *Adv. Synth. Catal.* 2014, *356*, 17–117; Glorius, F.; *et al.* *Nat. Chem.* 2013, *5*, 369–375; Itami, K.; *et al.* *Angew. Chem. Int. Ed*. 2012, *51*, 8960–9009; Ackermann, L. *Chem. Rev.* 2011, *111*, 1315–1345).

**Figure:**
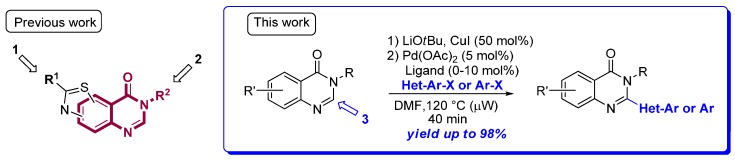


We described the first extensive study of palladium-catalyzed direct C–H (hetero)-arylation of quinazolin-4-ones with aryl iodides, bromides, and chlorides under microwave irradiation (Laclef, S.; *et al.* *Org. Lett.* 2015, *17*, 1700–1703; Godeau, J.; *et al.* *Eur. J. Org. Chem.* 2015, 7705–7717). This innovative methodology tolerates a broad range of heteroaryl and aryl halides substituted by electronically different groups. The scope of substrates was extended to pyridinopyrimidin-4-ones. This method provides an efficient, versatile, and rapid access to 2-arylquinazolin-4-one backbone and will be extended to our thiazoloquinazolin-4-one derivatives.

**Acknowledgments:** This work has been partially supported by Rouen University, INSA Rouen, CNRS (Centre National de la Recherche Scientifique) and Labex SynOrg (Agence Nationale de la Recherche ANR-11-LABX-0029). We gratefully acknowledge the financial support from the MESR (Ministère de l’Enseignement Supérieur et de la Recherche), the “Région Haute-Normandie,” and the CRUNCH network (Centre de Recherche Universitaire Normand de Chimie). We also thank Anton Paar GmbH (Graz, Austria) for provision of single-mode microwave reactor (Monowave 300) and for technical support.

### 2.5. Complex of Zinc and Lectins from Seeds of Vigna radiata as a Potential Anti-Diabetic Agent (A006)

Chhaya Harihar Gadgoli *, Lalit Sali, Mahesh Abhyankar and Prachi Pathak

Saraswathi Vidya Bhavan's College of Pharmacy, Dombivli East, Thane, Maharashtra 421204, India

* Correspondence: chhayahgadgoli@gmail.com

Proteins and phytohemagglutinins from *Vigna* species have been known to possess α-amylase inhibitory activity. Therefore, it was thought worthwhile to evaluate the lectins from *Vigna radiata* for antidiabetic activity. The literature reveals that such studies have not yet been carried out. The principal object of this study was to evaluate antidiabetic activity of the lectins from *V. radiata*. Another goal of this study was the binding of the zinc with the lectins obtained from *V*. *radiata* and an evaluation of its antidiabetic activity.

The *V. radiata* seed found contain galactose specific lectin. The MBL-I (Mung Bean Lectin I) may be a tetrameric protein with a molecular weight of 160–180 kDa and may be composed of an identical or nearly identical relative subunit with a molecular weight of 45–50 kDa. Amino acid analysis of purified mung bean lectin by reverse phase HPLC revealed that it contains glutamate in the highest proportion, followed by aspartate and histidine, which indicates that it has good zinc-binding potential. Binding of the lectins with zinc improved the overall stability and efficacy of the lectins. The antidiabetic activity was evaluated in Wistar rats using an alloxan-induced diabetic model, and the studies indicated significant (*p* < 0.001) reduction in elevated sugar levels. It is probable that the mechanism of antidiabetic action may be insulinomimetic, as it was found to bind with insulin antibodies in an Western blotting analysis.

The lectin obtained from *V. radiata* seeds and zinc lectin complexes has good potential to be explored as a safe, natural antidiabetic agent acting with a controlled reduction in blood glucose levels. These findings indicate that the mung bean lectin and zinc lectin complexes have tremendous medicinal potential as a herbal antidiabetic drug which needs to be explored.

### 2.6. Analgesic and Anti-Inflammatory Activity of Diterpenoid Alkaloids Isolated from the Central Asian Species of Aconitum and Delphinium Plants (A007)

Firuza Tursunkhodjaeva * and Farkhad Dzhakhangirov

Institute of the Chemistry of Plant Substances, Uzbek Academy of Sciences, 77, Mirzo Ulugbek street, Tashkent, 100170, Uzbekistan

* Correspondence: ftm40438@gmail.com

In different countries in Europe, Asia and America, plants containing diterpenoid alkaloids have been used in folk medicine since ancient times. *Aconitum* and *Delphinium* plants (from the *Ranunculaceae* family) and their extracts are currently used in Eastern medicine as an antirheumatic, an analgesic, an anti-inflammatory, and other remedies. More than 50 species of *Aconitum* (300 worlwide) and 100 species of *Delphinium* (450 worldwide) grow in the territory of Former Soviet countries, including Russia, Central Asia, and Kazakhstan.

We investigated antinociceptive and anti-inflammatory activity of individual diterpenoid alkaloids with lycoctonine, heteratisine and perhydrophenantrene carbon skeletons isolated from *Aconitum* and *Delphinium* species that are widespread in Central Asia, and revealed 25 promising substances. Antinociceptive activity was investigated with conventional tests for displaying analgesics with central mechanisms of analgesia (hot plate) and peripheral mechanisms (acetic writhing, local anesthesia). Anti-inflammatory activity was studied with the rat formalin test.

Compared to the antinociceptive activity of investigated alkaloids and underlying mechanisms of their pharmacological action, we divided them by the following types: activators of potentially gated Na^+^ channels of neurons that cause a shifting of the threshold of Na+ currents towards membrane hyperpolarization and that destroy neuronal conductivity; blockers of potentially gated Na^+^ channels that cause inhibition of fast-intake Na+ currents without changing its activation threshold; and blockers of *N*-cholinoreceptors.

**Acknowledgments:** Salimov B.T., Sultankhodzaev M.N. and Usmanova S.K. (Institute of the Chemistry of Plant Substances, Uzbek Academy of Sciences), who provided the investigated chemical substances.

### 2.7. Ebola Virus Disease: Questions, Ideas, Hypotheses and Models (A009)

Francisco Torrens ^1,^* and Gloria Castellano ^2^

^1^ Institut Universitari de Ciència Molecular, Universitat de València, Edifici d’Instituts de Paterna, P. O. Box 22085, 46071 València, Spain

^2^ Departamento de Ciencias Experimentales y Matemáticas, Facultad de Veterinaria y Ciencias Experimentales, Universidad Católica de Valencia San Vicente Mártir, Guillem de Castro-94, 46001 València, Spain

* Correspondence: torrens@uv.es

How is Ebola virus disease (EVD) contagious? What happens at the host-pathogen interface? Why are certain viruses capable of jumping into new species? Genetic plasticity is key if the virus is to overcome a host immune attack. Double-stranded ribonucleic acid (dsRNA) triggers the release of cytokines. What is the possibility of an outbreak in Spain? How alert should a country such as Spain be? Does the Law of Labour Risks observe protocol? Does Spain have the equipment and experience needed to treat EVD patients? Why is it difficult to control determined infections or find vaccines against virus-caused diseases? Furthermore, must one be critical of the protocols followed by the Spanish Government? Here, the model of Ebola virus transmission dynamics is reviewed, with the aim of providing a broad sketch of the fundamental human-Ebola-virus biophysical forces that enable and constrain EVD. Akhtar *et al.* reported a model of Ebola evading the immune system. What were the factors affecting the emergence, rapid spread, and uncontrolled nature of the 2014 virus outbreak? How is EVD treated? How does the Ebola virus replicate? How have bats evolved to resist fatality in the face of the Ebola virus? Did Ebola in Zaire exist in the African landscape before 1976, or did it evade detection and documentation? How does Ebola evade the immune system? Provisional conclusions follow: (1) Little is known about how viruses interact with the human immune system; (2) transmission among humans occurs via the exchange of blood and bodily secretions.

**Acknowledgments:** F.T. is grateful for the support from the Spanish Ministerio de Economía y Competitividad (Project No. BFU2013-41648-P) and the EU ERDF.

### 2.8. Looking for a PET Tracer for Imaging Apoptosis (A010)

Michel Monclus ^1,^*, Benoît Menghini ^1^, Véronique Kruys ^2^, Nicolas Preyat ^2^, Simon Lacroix ^1^ and Serge Goldman ^1^

^1^ PET/Biomedical Cyclotron Unit and Department of Nuclear Medicine, Erasme Hospital, Université Libre de Bruxelles, route de Lennik 808, 1070, Belgium

^2^ Institute for Molecular Biology and Medicine Université Libre de Bruxelles (IBMM, ULB) Rue des Profs Jeener et Brachet 12, 6041 Gosselies, Belgium

* Correspondence: mmonclus@ulb.ac.be

In multicellular organisms, homeostasis is maintained by a balance between cell proliferation and apoptosis (programmed cell death). It is a physiological form of cell death responsible for the deletion of non-repairable damaged, mutated, or cells which have lost their function.

We describe the synthesis of a series of potential inhibitors of caspases from a modified aspartic acid residue (fluoromethylketone (fmk)). The addition to the entire series of either a 3-cyano-4-fluoro-benzoyl- pattern or of a 4-fluoro-2-thiazolamino pattern will subsequently allow the introduction of a PET isotope (^18^F).

In order to determine potential candidates, the inhibitory activity of these compounds was evaluated *in vitro* on a series of human T cells and compared to the z-VAD-fmk as a reference.

### 2.9. Augmenting the Efficacy of Antifungal Intervention via Chemo-biological Approaches (A011)

Jong H. Kim *, Kathleen L. Chan and Luisa W. Cheng

Foodborne Toxin Detection and Prevention Research Unit, Western Regional Research Center, USDA-ARS, 800 Buchanan St., Albany, CA 94710, USA

* Correspondence: jongheon.kim@ars.usda.gov

Mycotic infection is becoming a serious health problem since effective antifungal agents for control of pathogenic fungi, especially drug-resistant pathogens, are often very limited. Fungal resistance to antimycotic agents frequently involves mutations caused by environmental stressors. In fungal pathogens, stress signals resulting from oxidative, cell wall stress, *etc.*, are integrated into the upstream mitogen-activated protein kinase (MAPK) pathways that regulate genes countering the stress. It is noteworthy that mutations in the MAPK signaling system result in fungal tolerance to cell wall disrupting agents or phenylpyrrole.

**Figure:**
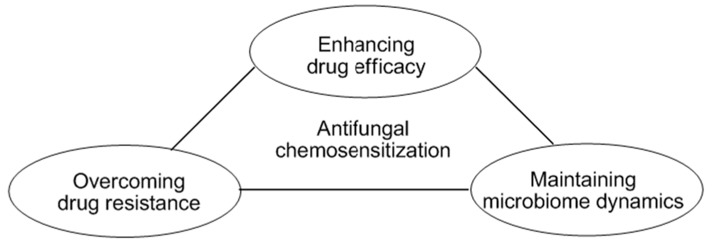


In a chemo-biological platform to achieve targeted antifungal intervention, the model yeast *Saccharomyces cerevisiae* served as a tool for identifying mechanisms of action of redox-active or cell wall disrupting agents. This also enabled the identification of new utility of known compounds or the utilization of natural products/derivatives as chemosensitizing agents to intensify the efficacy of conventional antimycotic agents (Campbell, B.C.; *et al. Front. Microbiol.* 2012, *3*, 79). Compounds targeting cellular antioxidant, mitochondrial, or cell wall integrity systems effectively inhibited the growth of pathogens and/or overcame fungal tolerance to antimycotic agents. Therefore, chemo-biological approaches lead to the development of novel intervention strategies, such as antifungal chemosensitization, which enhance the drug susceptibility of targeted fungi, and ensure the maintenance of healthy microbiome dynamics.

**Acknowledgments:** This research was conducted under the USDA-ARS CRIS Project 2030-42000-037-00D.

### 2.10. Application of RNA Aptamers to the Control of the Function of the Hepatitis C Virus-CRE Region (A012)

Alba Fernández-Sanlés ^1,†^, Beatriz Berzal-Herranz ^2,†^, Pablo Ríos-Marco ^2^, Cristina Romero-López ^2,^* and Alfredo Berzal-Herranz ^2,^*

^1^ Department of Experimental and Health Sciences, Universitat Pompeu Fabra, Parc de Recerca Biomèdica de Barcelona, Dr. Aiguader 88, Barcelona 08003, Spain

^2^ Institute of Parasitology and Biomedicine López-neyra, IPBLN-CSIC, PTS Granada, Av. Conocimiento s/n, 18016, Armilla (Granada), Spain

* Correspondence: aberzalh@ipb.csic.es (A.B.-H.); cristina_romero@ipb.csic.es (C.R.-L.)

^†^ These authors contributed equally to this work

Hepatitis C virus is an enveloped, ssRNA virus that infects 3% of the world’s population. No fully efficient therapy for treating hepatitis C exists. This is mainly due to the *quasi*-species structure of the RNA genome population, which favors the emergence of resistant viral variants. Despite the high variability rate, significant sequence and, more importantly, structure conservation can be found in the so-called functional genomic RNA domains, many of which have unknown roles for the consecution of the viral cycle. Such genomic domains are potential therapeutic targets. This study validates the use of RNA-based inhibitors (aptamers) as molecular tools to control the activity of the *cis*-acting replication element (CRE) within the HCV genome. The CRE is an essential partner for viral replication. Additionally, this structural domain is involved in the regulation of the protein synthesis. A set of 44 RNA aptamers was assayed for the ability to interfere with the viral RNA synthesis in a subgenomic replicon system. Four aptamers emerged as potent inhibitors of HCV replication by direct interaction with specific and well-defined functional RNA domains of the CRE, yielding a decrease in the HCV genomic RNA levels higher than 90%. Concomitantly, one of them also promoted a significant increase in viral translation (>50%), likely by its interaction with the nucleotides surrounding the viral stop translation codon. The three remaining aptamers efficiently competed with the binding of the NS5B protein to the CRE, thus explaining their antiviral activity. Present findings confirm the potential of the CRE as an anti-HCV drug target and support the use of aptamers as molecular tools for challenging the functionality of RNA domains in viral genomes.

**Figure:**
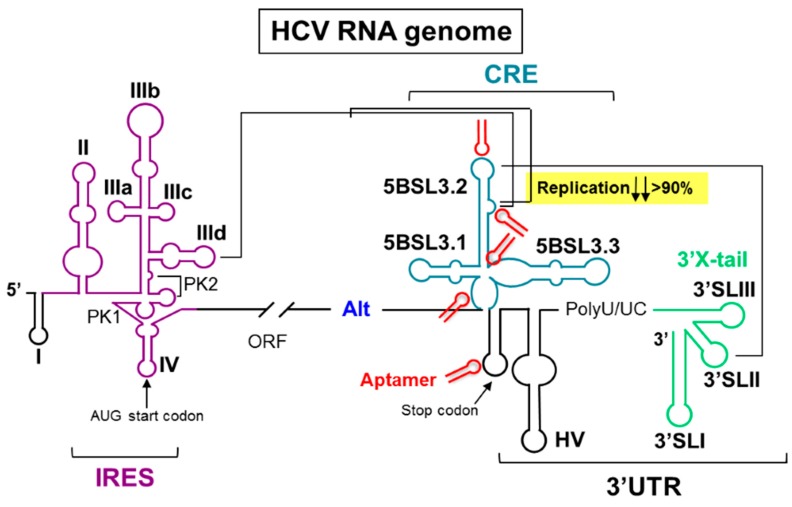


### 2.11. Inhibition of the Cancer Target Human Hyaluronidase Hyal‑1 by Natural Substances (A014)

Isabelle Lengers ^1^, Zoya Orlando ^1^, Matthias F. Melzig ^2^, Armin Buschauer ^3^, Andreas Hensel ^4^ and Joachim Jose ^1,^*

^1^ Institute of Pharmaceutical and Medicinal Chemistry, PharmaCampus, Westfälische Wilhelms-Universität, Corrensstraße 48, 48149 Münster, Germany

^2^ Institute of Pharmacy, Freie Universität Berlin, Königin Luise Str. 2+4, 14195 Berlin, Germany

^3^ Institute of Pharmacy, Department of Pharmaceutical/Medicinal Chemistry II, University of Regensburg, Universitätsstr. 31, 93040 Regensburg, Germany

^4^ Institute of Pharmaceutical Biology and Phytochemistry, PharmaCampus, Westfälische Wilhelms-Universität, Corrensstraße 48, 48149 Münster, Germany

* Correspondence: joachim.jose@uni-muenster.de

The negatively charged polysaccharide hyaluronic acid (HA) has diverse physiological and pathophysiological functions depending on its chain size. Space‑filling, anti‑inflammatory and antiangiogenic effects are triggered by high-molecular-weight HA (HMW HA) (>20 kDa). Hydrolyzation of HMW HA by Hyal‑1 results in low-molecular-weight HA (LMW HA) (<20 kDa), which leads to inflammatory and angiogenic effects (Stern, R. *Semin. Cancer Biol*. 2008, *18*, 275–280). For this reason, Hyal‑1 is an interesting target for drug discovery. The surface display of active Hyal‑1 on *Escherichia coli* via Autodisplay enables the screening for potential inhibitors in a whole cell system. Based on this technique, we determined the inhibitory effect of different natural substances on human Hyal-1. The IC_50_ values of the plant extracts *Malvae sylvestris flos*, *Equiseti herba* and *Ononidis radix* were determined to be between 1.4 and 1.7 mg/mL. Furthermore, the IC_50_ values of four triterpenoid saponines were determined. The obtained IC_50_ value for glycyrrhizinic acid, a known Hyal-1 inhibitor, was 177 µM. The IC_50_ values for the newly identified inhibitors gypsophila saponin 2, SA1641, and SA1657 were 108 µM, 296 µM, and 371 µM, respectively (Orlando, Z.; *et al.* *Molecules* 2015, *20*, 15449–15498). For the synthesis of new small molecule inhibitors targeting human Hyal-1, these extracts and natural compounds can be used as a starting point.

### 2.12. Synthesis and Pharmacological Properties of New GABA- and TRP Allosteric Modulators (A017)

Iryna Kravchenko, Alexandra Alexandrova, Elena Prokopchuk and Mariia Nesterkina *

Department of Pharmaceutical Chemistry, I.I. Mechnikov Odessa National University, 65026 Odessa, Ukraine

* Correspondence: mashaneutron@gmail.com, kravchenko.pharm@gmail.com

GABAergic and glycinergic systems are the targets of a wide range of drugs active on the CNS, including anxiolytics, sedative-hypnotics, general anesthetics and anticonvulsants (Macdonald, R.L.; *et al. Annu. Rev. Neurosci.* 1994, *17*, 569–602). Recent studies have reported that cyclic monoterpenes menthol and thymol also have actions within the CNS (Zhang, X.-B.; *et al. PLoS ONE* 2008, *3,* e3386) and act as a potent positive allosteric modulator of GABA_A_ receptors (Hall, A.C.; *et al. Eur. J. Pharmacol.* 2004, *506*, 9–16). It has been reported that salicylic acid derivatives activate heterologously expressed TRPA1 (Bandell, M.V.; *et al. Neuron* 2004, *41*, 849–857), a member of the TRP channel family expressed by nociceptors and its potential role as a sensor of noxious cold (Story, G.M.; *et al. Cell* 2003, *112*, 819–829; Bandell, M.V.; *et al. Neuron* 2004, *41*, 849–857). In this work, we present the synthesis and pharmacological investigation of some new menthol, thymol, and salicylic acid derivatives designed as GABA- and TRP allosteric modulators.

**Figure:**
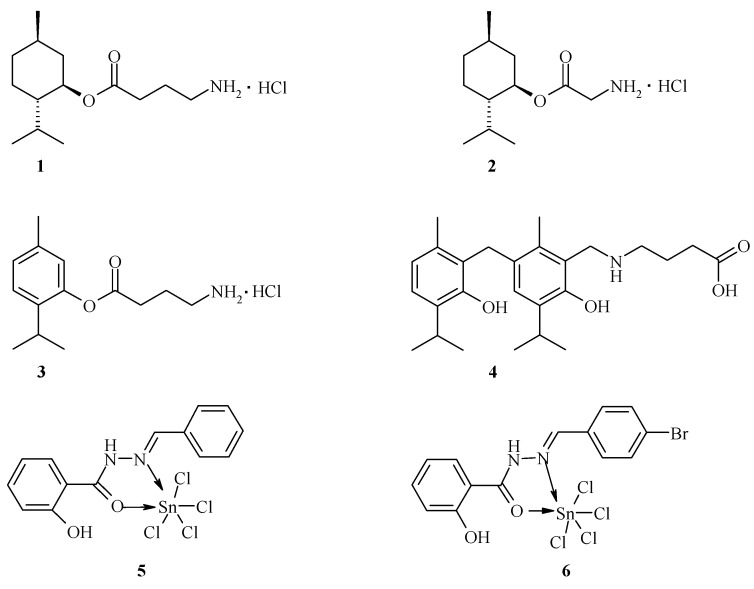


Our findings identified menthyl ester of GABA **1** (2-isopropyl-5-methylcyclohexyl 4-aminobutyrate) as a compound with anticonvulsant activity over a wide range of doses (87–1350 mg/kg), whereas menthyl ester of glycine **2** (2-isopropyl-5-methylcyclohexyl 2-aminoacetate) shows a significant sedative effect during the six hours after oral administration at 175 mg/kg. Furthermore, orally co-administered gidazepam (1 mg/kg) and compound **1** (175 mg/kg) produce synergistic effect in seizures prevention. Additionally, thymol ester of GABA **3** (20–100 mg/kg) as well as thymol derivative **4** (25–100 mg/kg) exhibits anti-seizure effects during the six hours after oral administration. Complex compounds obtained by SnCl_4_ interaction with salicyloylhydrazones (**5**, **6**) have demonstrated anti-inflammatory, anxiolytic, antidepressant and analgesic activities in different models *in vivo*. All aforementioned compounds can be considered as those that exhibit a combined pharmacological activity due to their simultaneous binding to different receptor types.

### 2.13. Potential Orally Active Heparin-Like Compounds: Synthesis and Anticoagulant Activity (A018)

Ana Rita Neves ^1^, Emília Sousa ^1,2^, Madalena Pinto ^1,2^ and Marta Correia-da-Silva ^1,2,^*

^1^ Laboratório de Química Orgânica e Farmacêutica, Departamento de Ciências Químicas, Faculdade de Farmácia da Universidade do Porto, 4050-313 Porto, Portugal

^2^ CIIMAR, Centro Interdisciplinar de Investigação Marinha e Ambiental, Universidade do Porto, 4050-123 Porto, Portugal

* Correspondence: m_correiadasilva@ff.up.pt

According to the World Health Organization, cardiovascular diseases are the most common cause of death worldwide. Although health has improved in the last several decades, lifestyle changes have led to an increased incidence of cardiovascular diseases. Currently, the available antithrombotic drugs are associated with significant drawbacks that limit their use, and the development of more advantageous drugs with less secondary effects is necessary. Our group discovered a new class of polysulfated small-molecules with anticoagulant and antiplatelet activities (Correia-da-Silva, M.; *et al. Eur. J. Med. Chem.* 2011, *46*, 347–2358; Correia-da-Silva, M.; *et al.* *J. Med. Chem.* 2011, *54*, 5373–5384). However, these polysulfated derivatives showed poor antithrombotic efficacy by *in vivo* oral administration in mice, predicted to be due to poor absorption in the gastrointestinal (GI) tract (Correia-da-Silva, M.; *et al.* *J. Med. Chem.* 2011, *54*, 5373–5384; Correia-da-Silva, M.; *et al. J. Med. Chem.* 2011, *54*, 95–106). The main aim of this work was to improve the oral bioavailability of these compounds. In order to get new optimized analogues, two strategies were considered: (i) obtaining conjugates with bile acids and (ii) introduction of a triazole ring.

Naringin-deoxycholic acid conjugate was obtained through a cross-linking reaction using 2-(1*H*-Benzotriazole-1-yl)-1,1,3,3-tetramethyluronium tetrafluoro borate (TBTU) as a coupling reagent. Triazole-linked xanthone glycoside was obtained through a copper(I)-catalyzed alkyne-azide cycloaddition followed by *O*- and *N*-deacetylation. Sulfation was successfully achieved with a triethylamine-sulfur trioxide adduct under microwave irradiation.

The three sulfated derivatives were screened for anticoagulant activity using the three classic clotting times: activated partial thromboplastin time (APTT), prothrombin time (PT), and thrombin time (TT). All the sulfated compounds prolonged the clotting times and the most active compound was the persulfated naringin-deoxycholic acid conjugate, exhibiting a double concentration value on the APTT (APTT_2_) in the micromolar range (around 44 µM). These new optimized analogues with anticoagulant activity are expected to cross the GI tract membranes after oral administration.

**Acknowledgments:** This research was partially supported by ERDF through the COMPETE and national funds through FCT, under the project PEst-C/MAR/LA0015/2013.

### 2.14. Click Chemistry for Advanced Drug Discovery Applications of Human Protein Kinase CK2 (A020)

Christian Nienberg ^1^, Anika Retterath ^1^, Kira Sophie Becher ^2^, Henning D. Mootz ^2^ and Joachim Jose ^1,^*

^1^ Institut für Pharmazeutische und Medizinische Chemie, PharmaCampus, Westfälische Wilhelms-Universität Münster, Corrensstraße 48, D‑48149 Münster, Germany.

^2^ Institut für Biochemie, Westfälische Wilhelms-Universität Münster, Wilhelm-Klemm-Straße 2, D‑48149 Münster, Germany.

* Correspondence: joachim.jose@uni-muenster.de

Human CK2 is a heterotetrameric constitutively active serine/threonine protein kinase and plays an important role in current cancer research (Trembley, J.H.; *et al. Biofactors* 2010, *36*, 187–195). The kinase is composed of two catalytic CK2α subunits and two regulatory CK2β subunits. Most protein–protein interaction (PPI) studies or screening assays are based on fluorescence detection and require the labeling of the target enzyme by a fluorophore. Unfortunately, through labeling by commercial applications, the catalytic subunit CK2α loses activity. Furthermore, the labeling ratio of the protein sample differs and is not exactly reproducible.

**Figure:**
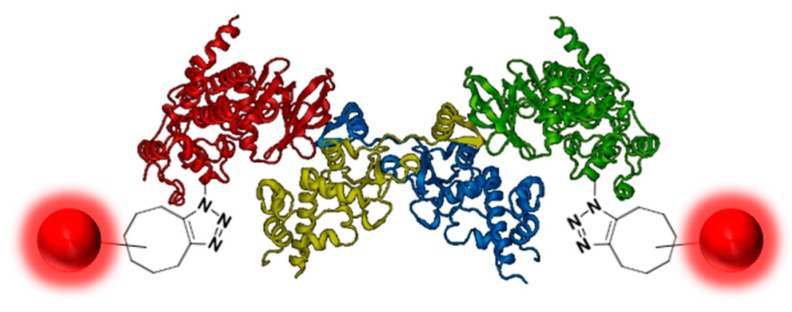
Modified from 4MD7 (PDB identification number)

The solution for this problem was a bioorthogonal click reaction of the protein kinase. By expanding the genetic code, the unnatural amino acid para-acidophenylalanine (pAzF) has been able to be incorporated into CK2 (Chin, J.W.; *et al. J. Am. Chem. Soc.* 2002, *124*, 9026–9027). Performing the SPAAC click reaction (Strain-Promoted Alkyne-Azide Cycloaddition) by the use of DBCO 545 (dibenzylcyclooctyne-fluor 545) has led to a specifically labeled human protein kinase CK2 (Mbua, N.E.; *et al. Chembiochem.* 2011, *12*, 1912–1921).

This site specific labeling does not impair the phosphorylation activity of the kinase, which was evaluated by capillary electrophoresis. The innovatively labeled kinase in combination with the Autodisplay technology could be a significant advancement for inhibitor screening assays by flow cytometry and for CK2α/CK2β interaction studies (Jose, J.; *et al. Microbiol. Mol. Biol. Rev.* 2007, *71,* 600–619).

### 2.15. Synthesis and Enantiomeric Purity Evaluation of a New Small Library of Promising Bioactive Chiral Derivatives of Xanthones (A021)

Carla Fernandes ^1,2,^*, Letícia Carraro ^1^, Ana Sofia Silva ^1^, Cláudia Veloso ^1^, Maria Elizabeth Tiritan ^1,2,3^, Artur M.S. Silva ^4^ and Madalena Pinto ^1,2^

^1^ Laboratório de Química Orgânica e Farmacêutica, Departamento de Ciências Químicas, Faculdade de Farmácia, Universidade do Porto, Rua Jorge Viterbo Ferreira nº 228, 4050-313 Porto, Portugal

^2^ Centro Interdisciplinar de Investigação Marinha e Ambiental (CIIMAR/CIMAR), Universidade do Porto, Rua dos Bragas 289, 4050-123 Porto, Portugal

^3^ CESPU, Instituto de Investigação e Formação Avançada em Ciências e Tecnologias da Saúde, Rua Central de Gandra, 1317, 4585-116 Gandra PRD, Portugal

^4^ Departamento de Química, Universidade de Aveiro, 3810-193 Aveiro, Portugal

* Correspondence: cfernandes@ff.up.pt

For the last several years, the search for new chiral derivatives of xanthones (CDX) with potential pharmacological properties has remained an area of interest of our group (Fernandes, C.; *et al.* *Bioorg. Med. Chem.* 2014, *22*, 1049–1062; Fernandes, C.; *et al. Eur. J. Med. Chem.* 2012, *55*, 1–11). Recently, we have described the ability of CDX to inhibit the growth of different human tumor cell lines (Fernandes, C.; *et al.* *Bioorg. Med. Chem.* 2014, *22*, 1049–1062). In fact, some of them exhibited interesting dose-dependent growth inhibitory effects being dependent on the stereochemistry.

Based on this work, we herein describe the synthesis of a new library of promising bioactive analogues in enantiomerically pure form, with good yields, short reaction times and no racemization. The optimization of the synthetic pathways to obtain the xanthonic derivative used as a chemical building block is also described.

The enantiomeric excesses for all synthesized CDX were measured by HPLC on (*S*,*S*)-Whelk-O1^®^ chiral stationary phase under polar-organic elution conditions, achieving values higher than 99%.

The evaluation of growth inhibitory activity on human tumor cell lines of new synthesized CDX is in progress.

**Acknowledgments:** This work was partially supported by the Strategic Funding UID/Multi/04423/2013 and UID/QUI/00062/2013 through national funds provided by the Foundation for Science and Technology (FCT) and the European Regional Development Fund (ERDF) in the framework of the programme PT2020 and the Portuguese NMR Network.

### 2.16. Synthesis of Aminated Xanthones: Exploiting Chemical Routes to Reach for Bioactive Compounds (A022)

Emília Sousa ^1,2,^*, Agostinho Lemos ^1^, Ana Gomes ^1,3^, Sara Cravo ^1^, Madalena Pinto ^1,2^

^1^ Department of Chemical Sciences, Laboratory of Organic and Medicinal Chemistry, Faculty of Pharmacy, University of Porto, 4050–313 Porto, Portugal

^2^ CIIMAR – Interdisciplinary Centre of Marine and Environmental Research, 4050–123, Porto, Portugal Portugal

^3^ Department of Biological Sciences, Laboratory of Microbiology, Faculty of Pharmacy, University of Porto, 4169–007 Porto, Portugal.

* Correspondence: esousa@ff.up.pt

Typically, about 90% of drug candidates are *N*-containing, and an even higher amount are *O*-containing. As a consequence, it is not surprising that alkylation and arylation of groups with nitrogen and oxygen emerge as major reactions to obtain bioactive compounds (Carey, J.S.; *et al. Org. Biomol. Chem.* 2006, 2337–2347). Xanthones are a class of *O*-heterocycles characterized by a dibenzo-γ-pyrone nucleus. This scaffold may be considered a “privileged structure” able to provide useful ligands for several types of receptors and/or enzymes targets by judicious structural modifications (Pinto, M.M.M.; *et al.* *Curr. Med. Chem.* 2005, *12*, 2517–2538). In our search for potential anticancer drugs, we pursued a hybridization approach of *N*-containing xanthones. *N*-Substitution is typically achieved by one of the following strategies: (i) direct reaction with alkyl-X or aryl-X or (ii) reductive alkylation using an appropriate aldehyde.

**Figure:**
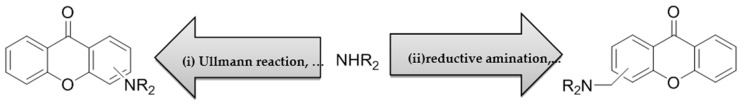


Herein, exploiting chemical routes by which bioactive *N*-containing xanthones can be reached are shared. The synthesis of new xanthone derivatives proceeds via both strategies, and the respective strengths and weaknesses are presented in a “medchem” perspective. Although chemical route (i) (S_N_2 reactions and nucleophilic aromatic substitutions) have provided interesting antitumor derivatives (Palmeira, A.; *et al., Biochem. Pharmacol.* 2012, *83*, 57–68), the reductive amination (ii) furnished a library of potential p53:MDM2 inhibitors with noticeable advantages, such as high-yield reactions, one-pot conversions, and aliphatic amines with low potential to form reactive metabolites.

The use of a variety of (thio)xanthone building blocks with various substituents and different reaction conditions allowed us to develop a repertoire of *N*-transformations, often referred to as the “chemist‘s toolbox” (Roughley, S.D.; *et al.* *J. Med. Chem.* 2011, *54*, 3451–3479).

**Acknowledgments:** This research was partially supported by ERDF through the COMPETE and national funds through FCT, under the project PEst-C/MAR/LA0015/2013.

### 2.17. Biomolecules and Natural Medicine Preparations: Analysis of New Sources of Bioactive Compounds from Ribes and Rubus spp. (A023)

Dario Donno*, Maria Gabriella Mellano, Alessandro K. Cerutti and Gabriele L. Beccaro

Department of Agriculture, Forestry and Food Science, University of Torino, 10124 Torino, Italy

* Correspondence: dario.donno@unito.it

It is well known that plants are important sources for the preparation of natural remedies, as they contain many biologically active compounds (Donno, D.; *et al. Vegetos* 2012, *25*, *21–29*). In particular, polyphenols, terpenic compounds, organic acids, and vitamins are the most widely occurring groups of phytochemicals (Donno, D.; *et al. J. Food Sci. Technol. Mysore* 2015, *18*, 1070–1085). Some endemic species may be used for the production of herbal preparations containing phytochemicals with significant bioactivity such as antioxidant activity and anti-inflammatory capacities, and other health benefits. Blackberry sprouts and blackcurrant buds are known to contain appreciable levels of bioactive compounds, including flavonols, phenolic acids, monoterpenes, vitamin C, and catechins, with several clinical effects (Donno, D.; *et al. J. Appl. Bot. Food Qual.* 2013, *86, 79–89*).

The aim of this research was to perform an analytical study of blackcurrant and blackberry bud-preparations in order to identify and quantify the main biomarkers, obtaining a specific phytochemical fingerprint to evaluate the single botanical class contribution to total phytocomplex and relative bioactivity, using a high-performance liquid chromatograph—diode array detector; the same analyses have been performed both in the university laboratory and commercially.

**Figure:**
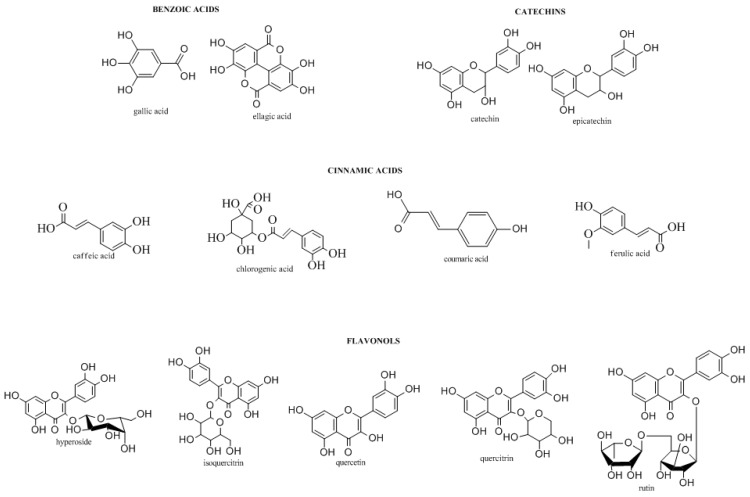


Different chromatographic methods were used to determine the concentrations of biomolecules in the preparations, allowing for the quantification of statistically significant differences in their bioactive compound content, both in the case of *Ribes nigrum* and *Rubus ulmifolius*.

Chemical, pharmaceutical, and environmental knowledge could be a useful tool for obtaining label certifications for the valorization of specific genotypes, with high clinical and pharmaceutical value (Donno, D.; *et al. Pharm. Biol.* 2013, *51*, *1282–1292*). This study allowed for the development of an effective tool for the natural preparation quality control and bioactivity evaluation through the chemical fingerprinting of bud preparations.

### 2.18. Two Methodologies in Molecular Structure and Intermolecular Interactions Analysis of Pharmaceuticals in Solid-state: X-Ray Diffraction and ^13^C CP/MAS NMR Data Mining (A024)

Dorota Maciejewska ^1,^*, Jerzy Żabiński ^1^, Mateusz Rezler ^1^ and Irena Wolska ^2^

^1^ Department of Organic Chemistry, Faculty of Pharmacy, Medical University of Warsaw, 1 Banacha Str., 02 097 Warsaw, Poland

^2^ Department of Crystallography, Faculty of Chemistry, Adam Mickiewicz University, 6 Grunwaldzka Str., 60 780 Poznań, Poland

* Correspondence: dmaciejewska@wum.edu.pl

Increasing demands from the pharmaceutical industry for the rapid molecular structure determination of pharmaceutical solids has prompted the development of X-ray diffraction and ^13^C CP/MAS NMR data analyses. The solid-state form of the drug can have a dramatic impact on the bioavailability, and the regulatory approval for many drugs is given only for the defined polymorph (Raw, A.; *et. al.* *Adv. Drug Deliv. Rev*. 2004, *56*, 397–414).

The intermolecular interactions are crucial in the interpretation of interactions between the biomolecules and macromolecular targets, and their analysis can provide essential information about how they occur.

The objective of the studies was to discuss the structures and the intermolecular interactions of the bis-nitriles **1**–**7** and the bis-amidines **6a**–**7a** in the solid-state.

**Figure:**
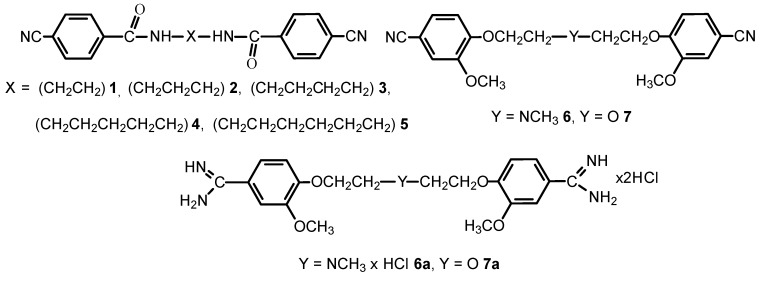


The compounds presented in this report can be considered as the pentamidine analogs, which are of interest because they have potential use as the chemotherapeutics against Pneumocystis pneumonia, as potent NMDA receptor inhibitors, or as anticancer and antimicrobial agents (Maciejewska, D.; *et al. Eur. J. Med. Chem*. 2012**,** *48*, 164–173; Berger, M.L.; *et al.* *Bioorg. Med. Chem*. 2015, *23*, 4489–4500).

We can conclude that the computations of shielding constants for isolated molecules together with the solid-state spectra are of considerable value in understanding the solid-state structures of pentamidine analogs. The structural information and intermolecular interactions in bis-nitriles are not transferable to the structural analysis for bis-amidines.

### 2.19. Study of a Series of 8-Substitued 7-Hydroxy-4-methylcoumarins as AChE and BuChE Inhibitors (A027)

Maria J. Matos ^1,^*, Fernanda Borges ^1^, Lourdes Santana ^2^, Eugenio Uriarte ^2^, Rosaria Medda ^3^, Adriana Murgia ^3^, Amalia Di Petrillo ^3^, Benedetta Era ^3^, Antonella Fais ^3^ and Francesca Pintus ^3^

^1^ CIQUP/Department of Chemistry and Biochemistry, Faculty of Sciences, University of Porto, 4169-007 Porto, Portugal

^2^ Department of Organic Chemistry, Faculty of Pharmacy, University of Santiago de Compostela, 15782 Santiago de Compostela, Spain

^3^ Department of Life Science and Environment, University of Cagliari, 09042 Monserrato, Cagliari, Italy

* Correspondence: mariacmatos@gmail.com

In the current work, we studied the interest of a series of 8-substitued 7-hydroxy-4-methylcoumarins as acetylcholinesterase (AChE) and butyrylcholinesterase (BuChE) inhibitors. For the best compounds of the series, the IC_50_ value was determined. This work was based on previous results and is a preliminary screening for further design and synthesis of new derivatives as potential compounds that can modulate enzymatic systems involved in the neurodegenerative diseases.

**Acknowledgments:** This work was partially supported by the University of Santiago de Compostela, University of Cagliari, Portuguese Foundation for Science and Technology (FCT) and QREN (FCUP-CIQ-UP-NORTE-07-0124-FEDER-000065), and FCT, POPH and QREN (SFRH/BPD/95345/2013).

### 2.20. Limiting the Number of Potential Binding Modes by Introducing Symmetry into Ligands: Structure-Based Design of Inhibitors for Trypsin-Like Serine Proteases (A028)

Norbert Furtmann ^1,2^, Daniela Häußler ^1^, Tamara Scheidt ^1^, Marit Stirnberg ^1^, Torsten Steinmetzer ^3^, Jürgen Bajorath ^2^ and Michael Gütschow ^1,^*

^1^ Pharmaceutical Institute, Pharmaceutical Chemistry I, Rheinische Friedrich-Wilhelms-Universität, An der Immenburg 4, Bonn 53121, Germany

^2^ Department of Life Science Informatics, B-IT, LIMES Program Unit Chemical Biology and Medicinal Chemistry, Rheinische Friedrich-Wilhelms-Universität, Dahlmannstrasse 2, Bonn 53113, Germany

^3^ Institute of Pharmaceutical Chemistry, Philipps University Marburg, Marbacher Weg 6, Marburg 35032, Germany

* Correspondence: guetschow@uni-bonn.de

In the absence of X-ray data, the exploration of compound binding modes continues to be a challenging task. For structure-based design, specific features of active sites in different targets play a major role in rationalizing ligand binding characteristics. For example, dibasic compounds have been reported as potent inhibitors of various trypsin-like serine proteases, the active sites of which contain several binding pockets that can be targeted by cationic moieties. This results in several possible orientations within the active site, complicating the binding mode prediction of such compounds by docking tools. Therefore, we introduced symmetry in bi- and tribasic compounds to reduce conformational space in docking calculations and to simplify binding mode selection by limiting the number of possible pocket occupations.

**Figure:**
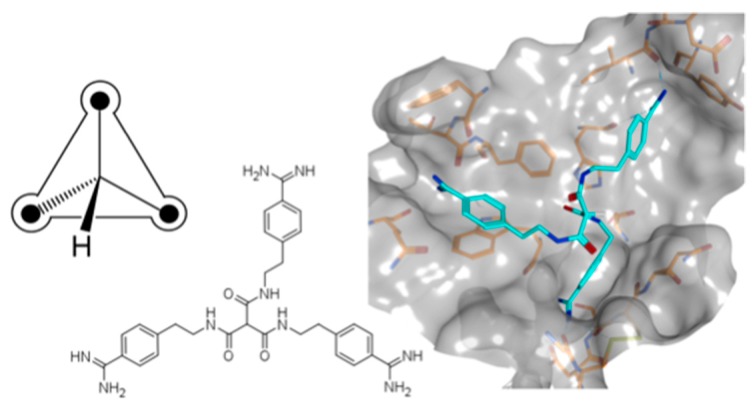


Herein, asymmetric bisbenzamidines were used as starting points for a multistage and structure-guided optimization. A series of compounds with either two or three benzamidine substructures was ultimately synthesized and evaluated as inhibitors of five serine proteases, leading to potent symmetric inhibitors for the pharmaceutical drug targets matriptase, matriptase-2, thrombin, and factor Xa (Furtmann, N.; *et al. Chem. Eur. J.* 2016, *22*, 610–625).

**Acknowledgments:** N.F. was supported by a fellowship from the Jurgen Manchot Foundation, Dusseldorf, Germany, and D.H. by a fellowship from the Bonn International Graduate School of Drug Sciences (BIGS DrugS). M.S. was supported by the German Research Foundation (STI 660/1-1) and the Maria von Linden-Program of the University of Bonn. The authors thank Dr. Matthias D. Mertens for support in building block synthesis and Dr. Eva Maurer and Anna-Madeleine Beckmann for providing matriptase-2.

### 2.21. Synthesis of N-{[5-Aryl/Alkyl-1,3,4-oxadiazol-2-yl]methyl}pyridin-2-amine as Antimicrobial and Anticancer Agents (A029)

Mohamed Jawed Ahsan * and Sunil Shastri

Department of Pharmaceutical Chemistry, Maharishi Arvind College of Pharmacy Ambabri Circle, Jaipur, Rajasthan 302 039, India

* Correspondence: jawedpharma@gmail.com

A new series of oxadiazole analogues was synthesized starting from 2-aminopyridine. The compounds were characterized by infrared (IR), nuclear magnetic resonance (NMR) and mass spectral analyses followed by their anticancer and antimicrobial activities. Three compounds were tested for *in vitro* anticancer activity against NCI-60 human cell lines of nine different panels including leukemia, non-small lung cancer, colon cancer, CNS cancer, melanoma, ovarian cancer, renal cancer, prostate cancer, and breast cancer, according to the National Cancer Institute (NCI US) Protocol at 10 µM (Boyd, M.R.; *et al.* *Drug Dev. Res.* 1995, *34*, 91–109; Monk, A.; *et al.* *J. Nat. Cancer Inst.* 1991, *83*, 757–766; Shoemaker R.H. *Nat. Rev. Cancer*, 2006, *6*, 813–823). The compounds *N*-{[5-(4-chlorophenyl)-1,3,4-oxadiazol-2-yl]methyl}pyridin-2-amine (**1c**), *N*-{[5-(4-methoxyphenyl)-1,3,4-oxadiazol-2-yl]methyl}pyridin-2-amine (**1f**) and *N*-{[5-(3,4-dimethoxyphenyl)-1,3,4-oxadiazol-2-yl]methyl}pyridin-2-amine (**1g**) showed anticancer with higher selectivity towards HOP-92 (non-small cell lung cancer). *N*-{[5-(4-Fluorophenyl)-1,3,4-oxadiazol-2-yl]methyl}pyridin-2-amine (**1b**) showed maximum antibacterial activity with a minimum inhibitory concentration (MIC) of 4–8 µg/mL, while *N*-{[5-(4-methoxyphenyl)-1,3,4-oxadiazol-2-yl]methyl}pyridin-2-amine (**1f**) showed maximum antifungal activity with MIC 4 µg/mL.

**Figure:**
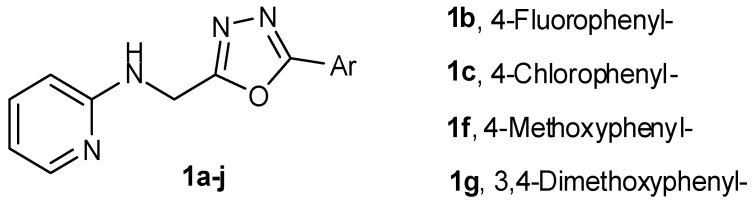


**Acknowledgments:** This work was partially supported by Department of Science and Technology (DST), Rajasthan (1156/2015). The authors are thankful to the National Cancer Institute (NCI), US for carrying out *in vitro* anticancer activity.

### 2.22. Evaluation of Alkanediamide-Linked Bisbenzamidines as Potential Antiparasitic Agents (A032)

Jean Jacques Vanden Eynde ^1^, Annie Mayence ^2^, Madhusoodanan Mottamal ^3^, Cyrus J. Bacchi ^4^, Nigel Yarlett ^4^, Marcel Kaiser ^5^, Reto Brun ^5^ and Tien L. Huang ^3,^*

^1^ University of Mons-Umons, department of organic chemistry, B-7000 Mons, Belgium

^2^ Haute Ecole Provinciale de Hainaut Condorcet, B-7730 Saint-Ghislain, Belgium

^3^ Xavier University of Louisiana, College of Pharmacy, New Orleans, LA 70125, USA

^4^ Pace University, Haskins Laboratories, New York, NY 10038, USA

^5^ Parasite Chemotherapy, Swiss Tropical institute, 4051 Basel, Switzerland

* Correspondence: thuang@xula.edu

A series of 15 alkanediamide-linked bisbenzamidines and related analogs was synthesized and tested *in vitro* against two *Trypanosoma brucei* (Tb) strains: *T. b. brucei* (Tbb) and *T. b. rhodesiense (*Tbr*)*; two *Plasmodium falciparum (*Pf*)* strains: a chloroquine-sensitive strain (NF54) and a chloroquine-resistant strain (K1); *Trypanosoma cruzi (*Tc*)*; and *Leishmania donovani (*Ld*)*. The *in vitro* cytotoxicity was determined against rat myoblast cells (L6). Seven compounds showed high potency toward both strains of Tb and Pf with the inhibitory concentrations for 50% (IC_50_) in the nanomolar range (IC_50_ = 1–96 nM). None of the tested derivatives was significantly active against Tc or Ld. Three of the more potent compounds were evaluated *in vivo* in mice infected with the drug-sensitive (Lab 110 EATRO and KETRI 2002) or drug-resistant (KETRI 2538 and KETRI 1992) clinical isolates of *T. brucei*. Compounds **1** and **2** were highly effective in curing 100% mice infected with the drug-sensitive strains, including a drug-resistant strain KETRI 2538, but were ineffective against KETRI 1992. Thermal melting of DNA and molecular modeling studies indicate AT-rich DNA sequences in the minor grove as possible binding sites for these compounds.

**Figure:**
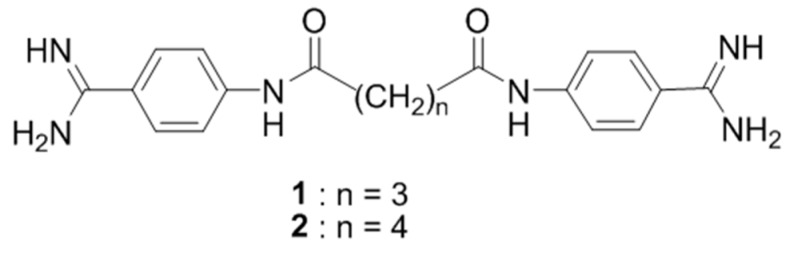


### 2.23. Synthesis, Anticancer Activity, and Molecular Docking Studies of Newer Quinoline Analogues (A033)

Mohamed Jawed Ahsan ^1,^*, Rita Yadav ^1^ and Surender Singh Jadav ^2^

^1^ Department of Pharmaceutical Chemistry, Maharishi Arvind College of Pharmacy Ambabri Circle, Jaipur, Rajasthan 302 039, India

^2^ Department of Pharmaceutical Chemistry, Birla Institute of Technology, Mesra, Ranchi, Jharkhand 835 215, India

* Correspondence: jawedpharma@gmail.com

A series of new quinoline analogues was prepared in two steps. All the synthesized compounds were characterized by IR, NMR, and mass spectral data. The anticancer activity was carried out as per the standard protocol, and LC_50_, TGI, and GI_50_ were calculated (Vichai, V.; *et al.* *Nat. Protoc*. 2006, *1*, 1112–1116; Prabhakaran, V.; *et al.* *Int. J. Tumor The*. 2014, *3*, 1–9). 1-(7-Hydroxy-4-methyl-2-oxoquinolin-1(2*H*)-yl)-3-(4-methoxylphenyl)-urea (**1**) showed maximum anticancer activity with a GI_50_ of 35.1 µM against HeLa (cervix cancer cell line) and 60.4 µM against MDA-MB-435 (breast cancer cell line), respectively. A molecular docking study implying epidermal growth factor receptor tyrosine kinase (EGFR-TK) was carried out to observe the binding mode of new quinoline analogues on the active site of EGFR-TK. Compound **1** showed a maximum docking score among the series of compounds. The amino acid residues Met793 showed backbone H-bonding with the hydroxyl group, while Asp855 showed side chain H-bonding with aryl NH group.

**Figure:**
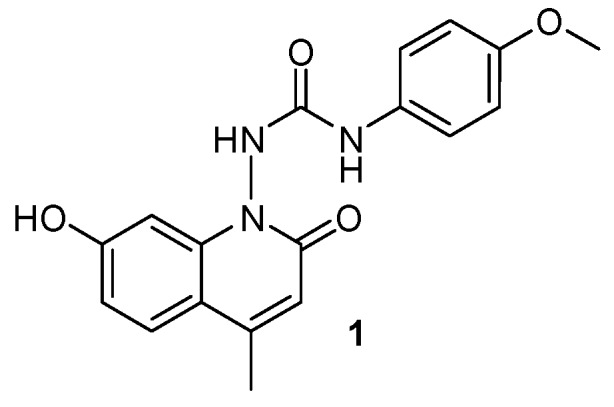


**Acknowledgments:** This work was partially supported by the Department of Science and Technology (DST), Rajasthan (1156/2015). The authors are thankful to the Anticancer Drug Screening Facility (ACTREC), Navi Mumbai, India for carrying out *in vitro* anticancer activity.

### 2.24. Identification of a Hit in a Small Library of Potential Antiplasmodial Imidazo[4,5-b]pyridines (A034)

Honoré Térence ^1^, Annie Mayence ^2^, Tien L. Huang ^3^ and Jean Jacques Vanden Eynde ^1,^*

^1^ University of Mons-UMons, Department of Organic Chemistry, B-7000 Mons, Belgium

^2^ Haute Ecole Provinciale de Hainaut Condorcet, B-7730 Saint-Ghislain, Belgium

^3^ Xavier University of Louisiana, College of Pharmacy, New Orleans, LA 70125, USA.

* Correspondence: jean-jacques.vandeneynde@ex.umons.ac.be

Recently, we have demonstrated that some bis(oxyphenylene)benzimidazoles constituted potential antiplasmodial candidates (Mayence, A.; *et al. Bioorg. Med. Chem.* 2011, *19*, 7493–7500), but they are characterized by a high lipophilic character. To circumvent that drawback, two series of structural analogs have been prepared. In the first series, oxygen atoms have been introduced in the linker separating both pharmacophores. In the second series, the heterocyclic skeletons have been replaced by imidazo[4,5-b]pyridine moieties. The antiplasmodial activity of the newly synthesized compounds has been evaluated against the chloroquine-sensitive strain NF-54. Their cytotoxicity in the presence of L6 rat skeletal muscle cells has also been determined.

Among the active derivatives, 2,2′-[propane-1,3-diylbis(oxy-1,4-phenylène)]bis-1*H*-imidazo[4,5-b]pyridine emerged as the most promising hit.

**Figure:**
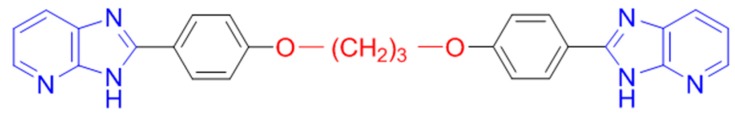


### 2.25. Search for Potent and Selective Aurora A Inhibitors Based on General Ser/ThrKinases Pharmacophore Model (A036)

Natalya I. Vasilevich *, Elena A. Aksenova, Denis N. Kazyulkin and Ilya I. Afanasyev

Novie Nauchnie Tekhnologii Ltd. (ASINEX company group), 20 Geroev Panfilovtsev Str., Moscow 125480, Russia

* Correspondence: nvasilevich@asinex.com

Based on the data of compounds known from the literature to be active against various types of Ser/Thr kinases, a general pharmachophore model for these types of kinases was developed. Search for the molecules fitting to this pharmacophore among ASINEX proprietary library revealed a number of compounds, which were tested and appeared to possess some activity against such Ser/Thr kinases as Aurora A, Aurora B, and Haspin.

Our work on optimization of these molecules to Aurora A kinase allowed us to achieve several hits in the 3–5 nM range of activity, with rather good selectivity and ADME properties.

Thus, we showed the possibility of performing the fine-tuning of the general Ser/Thr pharmacophore designed for desired types of kinase to get active and selective compounds.

**Acknowledgments:** The authors gratefully acknowledge support from the Ministry of Education and Science of the Russian Federation for funding (agreement 14.576.21.0019 dated July, 27, 2014).

### 2.26. Active Site Mapping of Human Cathepsin F with Dipeptide Nitrile Inhibitors (A038)

Janina Schmitz ^1,2^, Nobert Furtmann ^1,3^, Moritz Ponert ^1^, Ulrike Bartz ^2^, Jürgen Bajorath ^3^ and Michael Gütschow ^1,^*

^1^ University of Bonn, Pharmaceutical Institute, Pharmaceutical Chemistry I, An der Immenburg ^4^, Bonn 53121, Germany

^2^ Bonn-Rhein-Sieg University of Applied Sciences, von-Liebig-Str. 20, Rheinbach 53359, Germany

^3^ Department of Life Science Informatics, B-IT, Dahlmannstr. 2, Bonn 53113, Germany

* Correspondence: guetschow@uni-bonn.de

Cysteine cathepsins are lysosomal cysteine proteases which play roles in many physiological processes. Cathepsin F is predominantly expressed in macrophages. Major histocompatibility complex class II molecules (MHC-II) are expressed by antigen-presenting cell types including macrophages, B cells, and dendritic cells. The cleavage of the invariant chain is the key event in the pathway of MHC-II complexes. Cathepsin S was described as the major processing enzyme of the invariant chain, but it was shown that cathepsin F can adopt its role in cathepsin S deficient mice (Shi, G. P.; *et al.* *J. Exp. Med.* 2000, *191*, 1177–1186). Low molecular weight inhibitors for cathepsin F have not been investigated so far. We have chosen the dipeptide nitrile chemotype to develop covalent-reversible inhibitors for this target (Frizler, M.; *et al.* *Curr. Top. Med. Chem.* 2010, *10*, 294–322).

**Figure:**
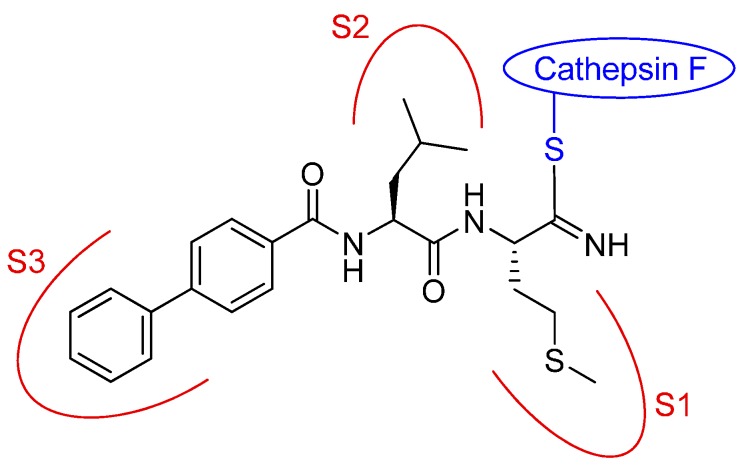


An active site mapping with a library of 52 nitrile-based cathepsin inhibitors was performed at human cathepsin F to draw structure-activity relationships. With the kinetic data in hand, new compounds with optimized residues in the P1, P2, and P3 positions were synthesized and evaluated. With all dipeptide nitriles including the newly synthesized derivatives, a 3D activity landscape was generated to visualize similarity-activity relationships of this series of cathepsin F inhibitors (Schmitz, J.; *et al.* *ChemMedChem* 2015, *10*, 1365–1377).

**Acknowledgments:** J.S. was supported by the Gender Equality Center of the Bonn-Rhein-Sieg University of Applied Sciences, St. Augustin, Germany, and N.F. by a fellowship from the Jürgen Manchot Foundation, Düsseldorf, Germany. The authors thank Tianwei Li, Adela Dudic, Karina Scheiner, Erik Gilberg, and Robert Sellier for assistance.

### 2.27. Synthesis and Biological Determination of a New Anthracen-9,10-dione Derivative as a Human CK2 Inhibitor (A042)

Samer Haidar *, Annika Meyers, Andre Bollacke and Joachim Jose

Institut für Pharmazeutische und Medizinische Chemie, PharmaCampus, Westfälische Wilhelms-Universität Münster, Corrensstr. 48, 48149 Münster, Germany.

* Correspondence: shaid_01@uni-muenster.de

Casein kinase 2 (CK2) is a ubiquitous kinase protein emerging as a target for several human diseases including cancer. Several active CK2 inhibitors have been developed in the last few years; most of them have an ATP-competitive type of inhibition, and only one inhibitor is in a clinical trial as an anticancer drug. Here, we report on the synthesis of two derivatives of 2,6-diaryl-anthracene-9,10-dione. One of them, 2,6-di(furan-3-yl)anthracene-9,10-dione compound 3, turned out to be active towards CK2 and ATP-competitive with an IC_50_ value of 2.35 µM and a K_i_ value of 1.26 µM. Molecular modeling studies were performed using MOE to explain the binding affinity of compound **1** in comparison to emodin. These indicated that, unlike emodin, compound **3** was not able to perform a hydrogen bond with Lys68, although the compound fits well in the active site of human CK2α, which explains the difference in the measured affinity between those two compounds (Haidar, S.; *et al.* *Pharmazie* 2015, *70*, 772–776).

**Figure:**
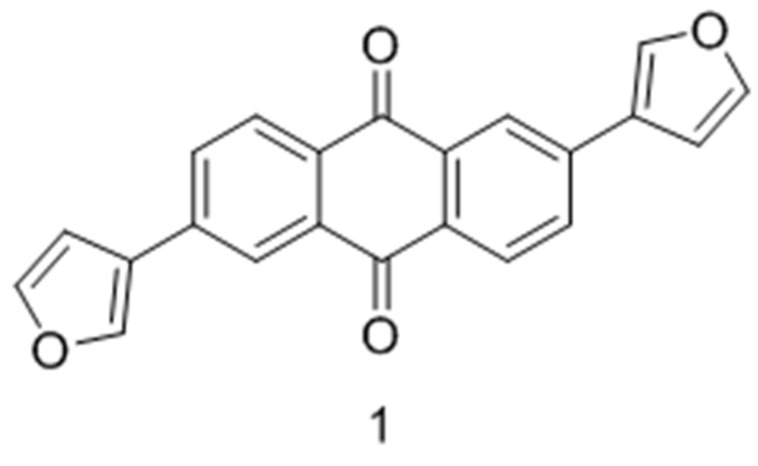


### 2.28. Spectroscopic Biosensors (A045)

Emmanuel Gosselin ^1,2,^*, Arnaud Petit ^1^, Joséphine Conti ^1^ and Joël De Coninck ^1,2^

^1^ Laboratory of Surface and Interfacial Physics, University of Mons, Place du Parc 20, Mons 7000, Belgium

^2^ Biosciences Institute, University of Mons, avenue Maistriau 19, Mons 7000, Belgium

* Correspondence: Emmanuel.gosselin@umons.ac.be

Sensors based on the molecular recognition of biomolecules have already attracted intensive interest in many different fields. Different surface sensitive techniques can be applied to detect these biomolecular interactions. We proposed an assessment of the utility of Fourier transform infrared (FTIR) spectroscopy in studying biomolecules attachment to inorganic surfaces in a variety of biosensing applications. We have designed a new generic device suitable for the investigation of ligand–receptor interactions based on the successive grafting of a novel silanization reagent and a bifunctional molecular clip directly at the surface of an internal reflection element.

**Figure:**
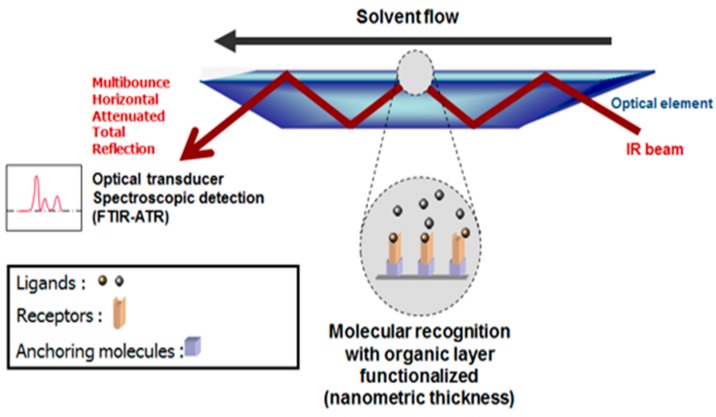


These molecular constructions lead to an activated transducer substrate ready for the covalent binding of any bioreceptor molecules. Contrary to SPR or quartz crystal microbalance (QCM) sensors, FTIR sensors provide useful spectroscopic information concerning the chemical nature of the interacting molecules and the amount of bound receptors and ligands. It is even possible that conformational transitions of the receptor during the interaction with the ligand can also be monitored. Currently, this information is not usually accessible using standard sensors that are limited to measuring physical modifications on the surface. We will illustrate the attachment of biomolecules to such organic surfaces through various systems commonly used in the biosensing field.

**Acknowledgments:** This work was financially supported by the F.R.S.-FNRS, CDR J.0116.14. We thank J.J. Vanden Eynde for helpful scientific discussions.

### 2.29. A Study of the Peculiarities of Virus Interactions and the Effectiveness of Antiviral Drugs in the Model of Mixed Infection (A046)

Liubov Biliavska *, Olga Povnitsa, Svitlana Zagorodnya, Sergei Voychuk, Liubov Zelena, Yulia Shamara and Nadiya Nesterova

Institute of Microbiology and Virology, National Academy of Sciences of Ukraine, Zabolotnogo str., 154, Kyiv, 03143, Ukraine

* Correspondence:bilyavskal@ukr.net1

Human adenovirus and herpes simplex virus cause infectious diseases of the eyes, respiratory, enteric, and urogenital tracts, and of the central and peripheral nervous system, are capable of persisting in a latent form, and are activated under the influence of endogenous and exogenous factors.

A high spread of infections caused by herpes virus and adenovirus, as well as their mixed infections, are a problems in modern medicine. During mixed infections, an absence of virus interaction as well as a mutual influence of viruses may originate.

The search for drugs that have relatively high activity against co-associated viruses through the inhibition of their reproduction and transmission is an important task. The activity of antiviral drugs under the condition of co-infection is not studied enough. Change in the nature of pathological processes were found as a result of the interference of the viruses and of the differences between drug activities against the co-associated viruses in conditions of the mono and mixed infections of MDBK cells. Both an increase in, and an inhibition of, drug activities were detected, which may lead to the formation of resistant strains of viruses.

### 2.30. Synthesis and Biological Evaluation of a New Thiazolo[5,4-f]quinazolines as Serine/Threonine Kinases Inhibitors (A047)

Damien Hédou ^1^, Corinne Fruit ^1^, Anne-Sophie Casagrande ^2^, Laurent Désiré ^2^, Bertrand Leblond ^2^, Laurent Meijer ^3^ and Thierry Besson1 ^1,^*

^1^ Normandie Univ, COBRA, UMR 6014 & FR 3038; Univ Rouen. CNRS, IRCOF, 1 Rue Tesnière, 76821 Mont St Aignan Cedex, France

^2^ Diaxonhit, 65 boulevard Masséna, 75013 Paris, France

^3^ Manros Therapeutics, Centre de Perharidy, 29680 Roscoff, France

* Correspondence: thierry.besson@univ-rouen.fr

In our continuous efforts to prepare novel heterocyclic scaffolds that are able to modulate the activity of kinases in signal transduction, synthetic routes to functionalized thiazolo[5,4-*f*]quinazolines were particularly studied *(*Logé, C.; *et al.* *Eur. J. Med. Chem.* 2008, *43*, 1469–1477; Testard, A.; *et al.* *Bioorg. Med. Chem. Lett.* 2006, *16*, 3419–3423). The chemical highlight of this work was the use of Appel salt (4,5-dichloro-1,2,3-dithiazolium chloride) for the conception of a 6-amino-2-cyanobenzo[d]thiazole-7-carbonitrile derivative as a versatile molecular platform from 5-nitroanthranilonitrile. Thus, introduction of various aliphatic, aromatic, or amino substituents at position 8 was best achieved via one-pot DMFDMA-mediated cyclization (Deau E.; *et al.* *Tetrahedron Lett.* 2013, *54*, 3518–3521). Transformation of the carbonitrile group into various chemical functions (e.g., imidate, ester, and amidine) allowed for the efficient preparation of a library of novel thiazoloquinazoline derivatives (Foucourt A.; *et al.* *Molecules* 2014, *19*, 15546–15571; Foucourt A.; *et al.* *Molecules* 2014, *19*, 15411–15439).

**Figure:**
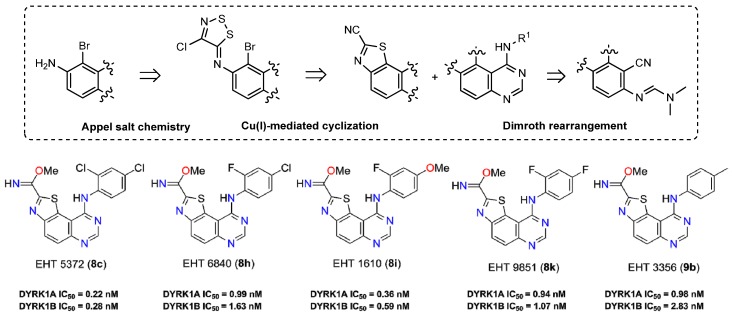


The biological results have identified great and selective inhibition against DYRK1A (subnanomolar range) and DYRK1B. (Courtadeur, S.; *et al.* *J. Neurochem*. 2015, 133, 440–451). The more active compounds are five methyl carbimidate derivatives exhibiting a great potential in the development of novel, highly potent dual inhibitors of DYRK1A and DYRK1B kinases that are involved in many neurodegenerative diseases (AD and other tauopathies), in genetic disease (DS), in oncology (Thompson, B.; *et al.* *J. Exp. Med.* 2015, *212*, 723–740), and in diseases involving abnormal pre-mRNA splicing.

**Acknowledgments:** This work has been partially supported by Rouen University and Labex SynOrg (ANR-11-LABX-0029). D.H. is grateful for financial support from the MESR (Ministère de l’Enseignement Supérieur et de la Recherche). We also thank Milestone s.r.l. (Bergamo, Italy) for provision of a multi-mode microwave reactor working at atmospheric pressure and for technical support.

### 2.31. Synthesis of Novel Alpha7-nAchR Ligands: From an Idea to in Rodent Results for Alzheimer’s [^18^F] TEP Imaging (A048)

Sylvain Routier

Univ Orleans, CNRS, Institut de Chimie Organique et Analytique, UMR 7311, BP 6759, F-45067 Orléans Cedex, France; sylvain.routier@univ-orleans.fr

The neurotransmitter acetylcholine (ACh) exerts its effects on the central nervous system (CNS) through two distinct muscarinic mAChRs and nicotinic nAChRs receptors types: nAChRs belong to the superfamily of ligand-gated ion channels, possessing a pentameric structure (Wu, J.; *et al.* *Int. J. Alzheimers Dis.* 2010, *2010*, 548913; Dajas-Bailador, F.; *et al.* *Trends Pharm. Sci.* 2004, *25*, 317–324; Gotti, C., *et al.* *Trends Pharm. Sci.* 2006, *27*, 482–491; Paterson, D., *et al.* *Prog. neurobiol*. 2000, *61*, 75–111). Because of their distribution and abundance in the CNS (in particular in the hippocampus and cortex), the a7 subtypes are potential diagnoses and therapeutic targets for brain disorders that involve these cerebral regions. In this field, α7 nAChR agonists were identified and allowed for the design of novel therapeutic agents for Alzheimer’s Disease (AD). Having in hand a human compatible [^18^F]-labeled positron emission tomography (PET) tracer to realize the early diagnostic or to validate the efficiency of therapies in clinical trials for AD is indubitably crucial.

With this aim, based on our expertise in heterocyclic bio-mimetic development (Neagoie, C.; *et al.* *Eur. J. Med. Chem.* 2012, *49*, 379–396; Boulahjar, R.; *et al.* *J. Med. Chem.* 2012, *55*, 9589–9606), we also endeavored to design novel α7 nAChR ligands and their transformation into a [18F] PET tracer. We synthesized a library of potent α7 nAChR ligands containing a quinuclidine, a tropane or a 8H-quinolizine moiety. We present herein chemistry (Routier, S.; *et al.* WO 2012143526 ; Pin, F.; *et al. Eur J. Med. Chem.* 2014, *83*, 214–224) SAR studies, molecular modeling docking studies, which confirmed the binding mode of the developed ligands, *in vitro* efficiency (SAR), radiolabeling, and *in vivo* results in rats.

**Acknowledgments:** the author is grateful to ANR Malz MInAlpha 7, the Labex Iron (ANR-11-LABX-18-01), the Région Centre (IMAD), the FEDER, and Cyclopharma Laboratories.

### 2.32. Floating Drug Delivery Systems with Xanthan Gum, Eudragit-RS PO or Lubritose SD: Nizatidine and Piracetam as Model Drugs (A049)

Azhidhack Hadjipour, Rena-Jean Palmer, Mohamed Zarara and Amal Ali Elkordy*

Sunderland School of Pharmacy, Faculty of Applied Sciences, University of Sunderland, Sunderland, SR1 3SD, UK

* Correspondence: amal.elkordy@sunderland.ac.uk

The aims of this study were to prepare and investigate the dissolution and floatability profiles of Nizatidine and Piracetam effervescent floating tablets and to study the effect of xanthan gum, Eudragit-RS PO, or Lubritose SD on tablet compression properties with or without granulation of the powder admixtures. Sodium bicarbonate was used to release CO_2_ when tablets come in contact with the acidic medium. Tablets without drugs were characterized for their floatability properties in a simulated gastric fluid (SGF) without enzymes at 37 °C. The successful formulations regarding floatability were incorporated with Nizatidine (50 mg/tablet) or Piracetam (30 mg/tablet). The powder admixtures were characterized for flow properties, and tablets containing drugs were evaluated via British Pharmacopeia quality control tests. All batches with Nizatidine that contain xanthan gum alone or in combination with Eudragit-RS PO showed good flow and compaction properties and also yielded significant (*p* < 0.05) swelling and floating results. However, Piracetam batches prepared with Lubritose SD showed poor compaction; therefore, granulation of the powders was applied to enhance floating tablet properties such as friability, floatability, and sustainability of the drug release of more than 6 hours. In conclusion, xanthan gum, Eudragit-RS PO (used with Nizatidine), and Lubritose SD (applied with Piracetam) could be promising excipients to formulate floating tablets.

### 2.33. Proteolysis Inhibitor E-Aminocaproic Acid as Effective Drug for Prevention and Treatment of Influenza, Other Acute Respiratory Viral Infections and Their Bacterial Complications (A050)

Viktor Lozitsky ^1,2^, Alla Fedchuk ^1,2^, Tetyana Grydina ^2,3^, Larysa Shytikova ^2^, Lyubov Mudryk ^2^, Lidiya Socheslo ^2^, Mikhailo Lebediuk ^1,3^, Oleksandr Voronkov ^4^, Volodymir Trykhlib ^5^, Arkadyi Frolov ^6^, Victoria Zadorozhna ^6^, Vira Buiko ^3^ and Sergyi Tkachuk ^7^

^1^ Research Center “Biomedical Testing of Preparations and Products”, Odessa 65003, Ukraine

^2^ I.I. Mechnikov Ukrainian Anti-Plague Research Institute, Ministry of Health, Odessa 65003, Ukraine

^3^ Odessa National Medical University, Odessa, 65000 Ukraine

^4^ State Enterprise “Research Enterprise Institute of Chemical Technology “Chemtechnology”, Sevtodonetsk 93400, Ukraine

^5^ Ukrainian Military Medical Academy, Kyiv 03049, Ukraine

^6^ L.V.Gromashevsky Institute of Epidemiology and Infectious Diseases of NAMS of Ukraine, Kyiv 03680, Ukraine

^7^ Military Medical Clinical Center of the Western Region, Lviv 79010, Ukraine

* Correspondence: lorevit@ukr.net

We have established the efficacy of proteolysis inhibitor (PI) aminocaproic acid (ACA) in viral infections as a result of its impact on the etiological factor and pathogenesis of the infection. The Ministry of Health of Ukraine, on the basis of our studies and clinical trials, allowed for the use of ACA for prophylaxix and treatment of influenza and other acute respiratory viral infections (ARVIs). Including ACA to therapeutic complex for the treatment of influenza and other ARVI in children and neonates led to a decrease in the duration of symptoms of intoxication, catarrhal phenomena, and fever as well as to a decrease in the number of complications. The prevention effectiveness of ACA has also been established. In light of the results, we recommend the use of ACA for the efficient prophylaxix of ARVIs and pneumonia in organized collectivities in any period of increased incidence of these infections. Our research has shown that the combined use of drugs with different mechanisms of action—PI ACA and neuraminidase inhinbitor Tamiflu—causes a synergetic effect. We also studied the antibacterial action of ACA on *S. aureus* strains with different sensitivity to antibiotics. ACA inhibits all these strains, and the combined use of ACA with antibiotics magnifies the antibacterial effect.

### 2.34. Quaternary Ammonium Sophorolipids as Renewable-Based Antimicrobial Products (A0051)

Elisabeth I. P. Delbeke ^1^, Bart I. Roman ^1^, Sophie L. K. W. Roelants ^2,3^, Inge N. A. Van Bogaert ^2^, Guy B. Marin ^4^, Kevin M. Van Geem ^4^ and Christian V. Stevens ^1,^*

^1^ SynBioC, Department of Sustainable Organic Chemistry and Technology, Ghent University, Coupure Links 653, 9000 Ghent, Belgium.

^2^ Department of Biochemical and Microbial Technology, Ghent University, Coupure Links 653, 9000 Ghent, Belgium

^3^ Bio Base Europe Pilot Plant (BBEU), Rodenhuizekaai 1, 9042 Ghent (Desteldonk), Belgium

^4^ LCT, Department of Chemical Engineering and Technical Chemistry, Ghent University, Technologiepark 914, 9052 Ghent, Belgium.

* Correspondence: Chris.Stevens@UGent.be

In the European chemical industry, there is a strong drive to shift from fossil to renewable resources in the pursuit of sustainability. Sophorolipids, a class of biosurfactants, are interesting renewable resources, since they combine a complex structure with divergent biological and physico-chemical properties. The microbially produced lactonic sophorolipids were used for the production of a broad range of innovative sophorolipid amines (**1**) and sophorolipid quaternary ammonium salts (**2**). These sophorolipid quaternary ammonium salts were evaluated for their antimicrobial activity against Gram-negative and Gram-positive bacterial test strains. Minimum inhibitory concentration (MIC) values were determined for the active compounds. Values of 5–8 µM results were obtained for the derivatives containing an octadecyl chain attached to the nitrogen atom, compared to values of 10–52 µM for the antibiotic gentamicin sulfate. These results show great promise for modified sophorolipids in the medical sector, for example, for the inhibition of biofilm formation.

**Figure:**
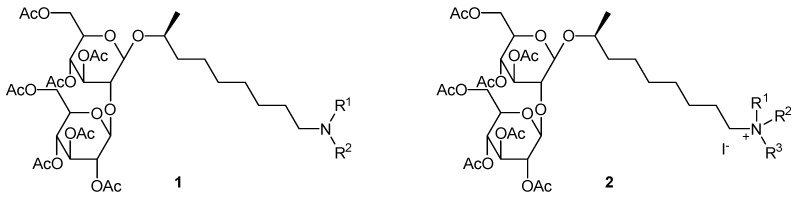


**Acknowledgments:** The research leading to these results received funding from the Long Term Structural Methusalem Funding by the Flemish Government (Grant number BOF09/01M00409). The authors gratefully acknowledge the company Ecover (Malle, Belgium) and the InBio research group (Department of Biochemical and Microbial Technology, Ghent University, Belgium) for the delivery of the sophorolipid starting compound, and the Laboratory for Microbiology (Ghent University, Belgium) for the evaluation of the antimicrobial activities. Bart I. Roman is a fellow of the fund for Scientific Research—Flanders (FWO-Vlaanderen).

### 2.35. The Transdermal Delivery Efficacy of Rimantadine under Experimental Influenza Model in Mice (A052)

Iryna Kravchenko ^1,^*, Viktor Lozitsky ^2,^*, Regina Lozytska ^2^ and Vitalyi Larionov ^1^

^1^ Odessa National University, Odessa 65000, Ukraine

^2^ Bogatsky Physico-Chemical Institute of NAS of Ukraine, Odessa 65080, Ukraine

* Correspondence: kravchenko.pharm@gmail.com; lorevit@ukr.net

Transdermal delivery of drugs is a novel method of pharmacotherapy for many diseases. The main advantages of this method of administration are:

- a prolonging of the drug action;
- an absence of the drug concentration hopping; and
- a decrease in the side effect risk.

This method of administration has to be viewed for preventive actions and therapy of viral respiratory infections. The aim of this work was to study the possibility of transdermal rimantadine delivery for influenza A prophylaxis and treatment in experimental animals. Rimantadine was administrated in the hydrogel matrix (formed from 1.2-propylene glycol and polyvinyl alcohol) in doses of 1–2 mg/mouse applied on the shaved back of mice one day before infection. Mice of experimental and control groups were infected intranasally with a highly pathogenic influenza virus A/PR/8/34 (H1N1). Challenge was carried out using for animals for each virus dilution within the range of 10–1 to 10–7. Deaths of animals were recorded for 14 days. The results of our investigations had shown high anti-influenza efficacy of rimantadine under transdermal delivery. Differences of LD50 between control and experimental groups consist 1.7–1.75 log10 when the dose of rimantadine was 1 mg/mouse, and 2.25 log10 when preparation was administrated in dose 2mg/mouse. In this study, the anti-influenza efficacy of rimantadine after its transdermal administration was established for the first time.

**Figure:**
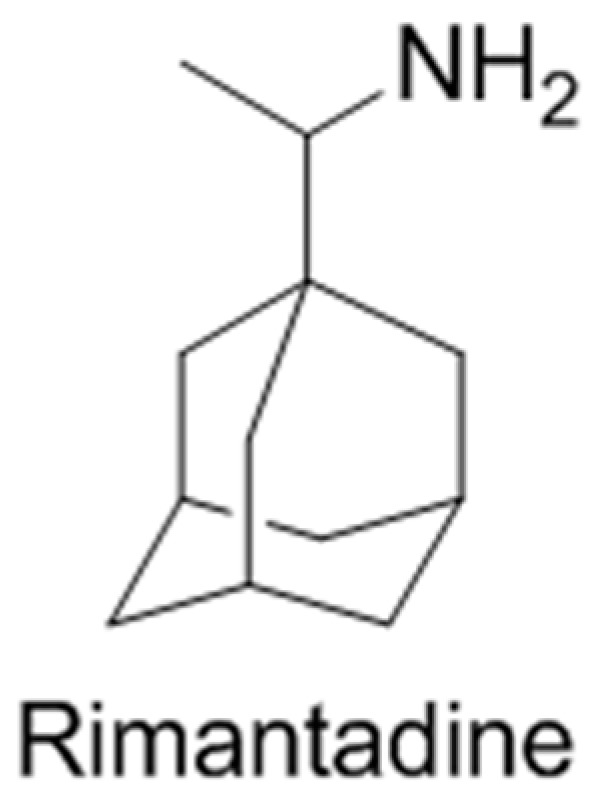


### 2.36. Arresting Cell Growth by Novel Functionalized Indolocarbazoles (A053)

Hannah J. Winfield, Kevin D. O'Shea and Florence O. McCarthy *

Department of Chemistry and ABCRF, University College Cork, Western Road, Cork, Ireland

* Correspondence: f.mccarthy@ucc.ie

Cancer causes about 13% of all human deaths and at least one fifth of all deaths in Europe and North America (GLOBOCAN 2008, database (version 1.2) http://globocan.iarc.fr). Although chemotherapy is increasingly prescribed, it is not without side effects; therefore, new, more selective remedies for cancer sufferers must be found.

Since the discovery of the anticancer properties of the indolocarbazole Staurosporine 1, many analogues have been synthesized in order to obtain compounds that have a higher potency with respect to anticancer mechanisms (Peifer, C.; *et al*. *J. Med. Chem.* 2006, *49*, 1271–1281; Pierce, L.T.; *et al., Tetrahedron* 2011, *67*, 4601–4611; Pierce, L.T.; *et al*. *Tetrahedron* 2010, *66*, 9754–9761).

**Figure:**
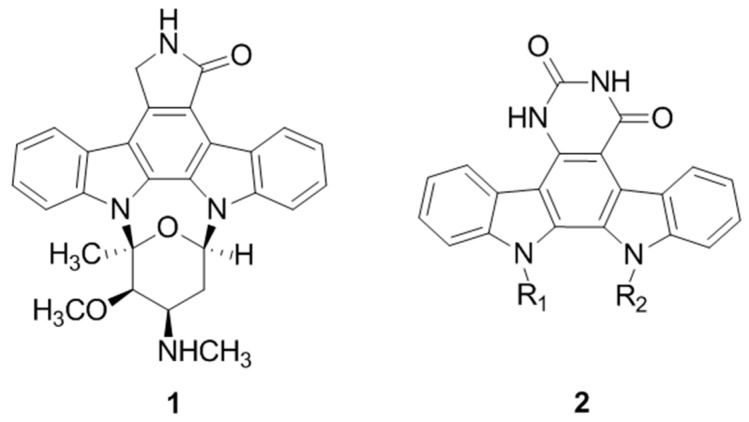


The overall objective of this project was to produce selective and highly potent novel anticancer agents through modification of the indolocarbazole structure, and one focus of this work was the replacement of the lactam/maleimide heretocycle to form a series of novel indolocarbazole derivatives including the first reported synthesis of a series of novel substituted indolocarbazole uracils (**2**) (Pierce, L. T.; *et al., Eur. J. Med. Chem.* 2012, 56, 292–300). Biological evaluation via the NCI 60 cell line screen has been completed for a number of these compounds, and some show significant selectivity towards individual leukemia and melanoma cell lines.

**Acknowledgments:** This work was funded by the Irish Research Council.

### 2.37. Heterocycles to Block the Cell Cycle: Novel Ellipticines and Their Anticancer Effects (A054)

Elaine Caitriona O’Sullivan, Mary L McKee and Florence O McCarthy *

Department of Chemistry and ABCRF, University College Cork, Western Road, Cork, Ireland

* Correspondence: f.mccarthy@ucc.ie

Ellipticine is a natural product possessing multimodal cytotoxic activity including DNA intercalation, topoisomerase II inhibition, c-Kit kinase inhibition, and restoration of function to mutant p53 protein (O’Sullivan, E.C.; *et al. Stud. Nat. Prod. Chem.* 2013, *39*, 189–232). While ellipticine itself is not a suitable candidate for therapeutic use, derivatives including 2-methyl-9-hydroxyellipticinium acetate and 2-(2-(diethylamino)ethyl)-9-hydroxyellipticinium chloride have progressed to clinical trials (Miller, C.M.; *et al. RSC Adv*. 2012, *2*, 8883–8918).

**Figure:**
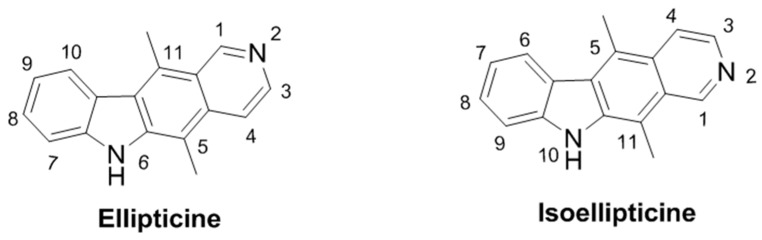


The effect of derivatization on the isoellipticine is uncharted and structural diversification of isoellipticine could lead to drug candidates with a better clinical profile due to enhanced target specificity. Our initial approach was to use substitutent modification at positions 10, 7 and 2 (salt formation at the N2 position represents a favorable attribute for cytotoxic activity, as illustrated by the two most successful ellipticines). A number of novel derivatives of isoellipticine have been synthesized and further derivatized. Preliminary biological testing of novel compounds was performed using a topoisomerase II decatenation assay and via assessment of the anticancer profile using the National Cancer Institute 60 cell line screen for cellular activity (Miller, C.M.; *et al*. *Org. Biomol. Chem*. 2012, *10*, 7912–7921; Deane, F.M., *et al. Org. Biomol. Chem*. 2013, *11*, 1334–1344; Russell, E.G.; *et al*. *Invest. New Drugs,* 2014, *32*, 1113–1122).

We present here the design, synthesis and anticancer properties, and significant cell line selectivity of a series of novel ellipticine derivatives devised in our laboratory.

**Acknowledgments:** This work was funded by the Irish Research Council.

## 3. Conclusions

The First International Electronic Conference on Medicinal Chemistry can be considered a success and effectively deserves the adjective “international,” since it gathered 206 authors from 18 different countries: Belgium, Bulgaria, Canada, France, Germany, India, Ireland, Italy, Japan, Poland, Portugal, Russia, Spain, Switzerland, Ukraine, United Kingdom, United States of America, and Uzbekistan. The website was visited by more than 25,000 individuals and dozens of comments have been posted, thus enabling the initiation of fruitful discussions and future collaborations. The award for the most viewed slide show during the month of November (>300 views) was given to the group of C. Nienberg, A. Retterath, K.S. Becher, H.D. Mootz, and J. Jose for the work entitled “Click Chemistry for Advanced Drug Discovery Applications of Human Protein Kinase CK2” (A020). Members of the scientific advisory committee elected as the best presentation the research topic of K. Zhu and M. Kai, entitled “Convenient Drug-Resistance Testing of HIV Mutants” (A030).

Thanks to the support of the organizers and the confidence of the participants, we are proud to announce that the Second International Conference on Medicinal Chemistry will be held in November 2016 on www.sciforum.net/conference/ecmc-2. All of us hope that you will have the opportunity to attend, as authors or visitors.

## Supplementary Materials

The full presentations are available online at www.sciforum.net/conference/ecmc-1.

